# Identification of human papillomavirus DNA gene sequences in human breast cancer

**DOI:** 10.1038/sj.bjc.6602778

**Published:** 2005-10-11

**Authors:** C-Y Kan, B J Iacopetta, J S Lawson, N J Whitaker

**Affiliations:** 1 School of Biotechnology and Biomolecular Sciences, University of New South Wales, Sydney, NSW 2052, Australia; 2 Department of Surgery, University of Western Australia, Nedlands, Australia

**Keywords:** human papilloma virus, human breast cancer, grade of tumour, patient mortality, hormone receptor status, abnormal p53 protein expression, p53 mutations and ERB-2 expression

## Abstract

Human papilloma viruses (HPVs) are accepted as being carcinogenic in human cervical and anogenital cancers. The suspicion that HPVs may also have a role in human breast cancer is based on the identification of HPVs in human breast tumours and the immortalisation of normal human breast cells by HPV types 16 and 18. For this investigation, DNA that had been previously extracted and fresh frozen at −70°C from 50 unselected invasive ductal breast cancer specimens were screened by polymerase chain reaction (PCR) for HPV type 16, 18 and 33 gene sequences. We show that HPV 18 gene sequences are present in DNA extracted from breast tumours in Australian women. Overall, 24 (48%) of the 50 samples were HPV positive. Overall no correlations with tumour grade, patient survival, steroid receptor status, ERB-2, p53 expression and mutation were observed. Human papilloma viruses may have a role in human breast cancer. We speculate that HPVs may be transmitted by hand from the female perineum to the breast.

It is accepted that human papillomavirus (HPV) types 16 and 18 are carcinogenic, and that probably HPV types 31 and 33 are also carcinogenic in human cervical and anogenital cancers (IARC, 1995). The suspicion that HPVs may also have a role in human breast cancer is based on the identification of HPVs in human breast tumours and the immortalisation of normal human breast cells by HPV 16 and 18 (Band *et al*, 1990; De Villiers *et al*, 2005).

Human papilloma virus 16 has been identified in breast tumours in Italian women and breast tumours in Norwegian women who had previous cervical neoplasia (Hennig *et al*, 1999). Human papilloma virus 33 has been identified in breast cancer in Chinese and Japanese women (Yu *et al*, 1999). Human papilloma virus 11, 16 and 18 have been identified in breast cancer in US and separately in Brazilian women (Liu *et al*, 2001; Damin *et al*, 2004). No HPVs have been identified in normal breast tissues from normal women who have had cosmetic surgery. No significant correlations have been observed between HPVs identified in human breast cancer and hormone receptor status of the tumour. Recently, de Villiers *et al* (2005) have identified a wide range of HPV types in cancer of the breast and nipples (De Villiers *et al*, 2005). In this latter investigation, the cancer of the nipple specimens had histological features consistent with human HPV infections. Other investigators have sought, but failed, to identify HPVs in human breast tumours. These various studies have been briefly reviewed by De Villiers *et al* (2005).

The aims of our investigation are to determine: (1) if HPV types 16, 18 and 33 gene sequences (the most common HPV carcinogens in cervical cancer) are present in breast cancers that have occurred in predominantly Caucasian Australian women, and (2) if there are any correlations between the presence of HPV sequences and grade of tumour, patient mortality, hormone receptor status, abnormal p53 protein expression, the presence of p53 mutations and ERB-2 expression.

## MATERIALS AND METHODS

For this investigation, DNA that had been previously extracted and fresh frozen at −70^O^C from 50 unselected invasive ductal breast cancer specimens were screened by polymerase chain reaction (PCR) for HPV type 16, 18 and 33 gene sequences. The DNA samples were amplified twice per sample using GenomiPhi™ DNA Amplification Kit (Amersham Biosciences). The DNA quality was confirmed by PCR, amplifying 268 bp of the *β*-globin gene. These samples were then screened for the presence of HPV by PCR using primers that could detect 140 bp in the E6 region of HPV 16, 18 and 33 (Yu *et al*, 1999). DNA extracted from cervical cancer cell lines HeLa and SiHa cells were used as positive controls for HPV 18 and 16, respectively. Plasmid plink 322 HPV 33 was used as a positive control for HPV 33. DNA from leukaemia Raji cells was used a negative control. The PCR products were separated on 7.6% PAGE and visualised by SYBR Green I (Molecular Probes). The screening was repeated five times using different batches of DNA samples amplified by the GenomiPhi™ DNA Amplification Kit (Amersham Biosciences). Human papilloma virus-positive samples were sequenced (there was sufficient material to sequence 18 of 24 HPV-positive samples).

Grade of tumour and survival of patients were known for each sample. Screening for exons 5–8 inclusive of the p53 gene were carried out using PCR–SSCP techniques as described and reported earlier by our group for this tumour series (Soong *et al*, 1997). Overexpression of p53 protein in primary breast tumours was investigated using immunohistochemistry with the DO7 monoclonal antibody as reported previously (Soong *et al*, 1997). Steroid receptor concentrations and ERB2 gene amplification were determined as previously described and reported (Soong *et al*, 1997).

## RESULTS

**Figure 1:**
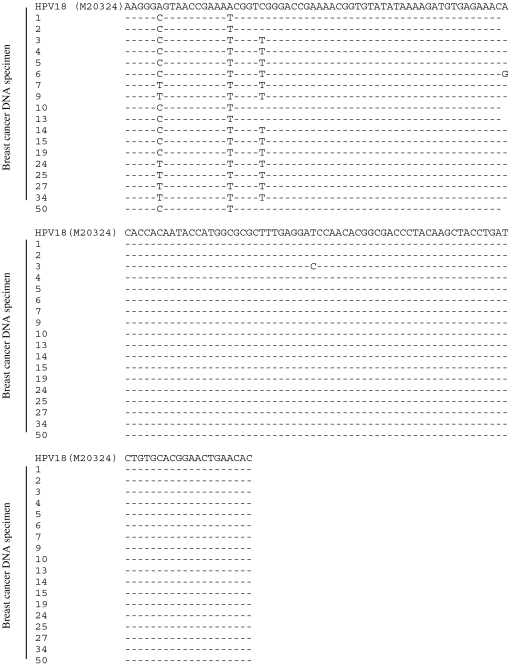
HPV DNA sequences. The DNA sequences of 18 HPV-positive samples were compared with the HPV 18E6 sequence (accession number: M20324) in the National Centre for Biotechnology Information database by the BLASTn program. Five variants of HPV 18 were identified.

Overall, 24 (48%) of the 50 samples were HPV positive. Sequencing of 18 unselected samples showed them to be variants of HPV 18 (there was insufficient material to sequence six of the 24 positive samples). In each separate PCR the percentage of breast cancer DNA samples that was HPV positive ranged from 2 to 32%. An added amplification of DNA before PCR and use of SYBR Green I for detection improved viral detection. As shown in Figure 1, there were five variants of HPV 18 sequences identified among the 18 sequenced samples. Such variations indicate that the presence of HPV 18 sequences is not due to contamination.

As shown in Table 1, there were no significant correlations between grade of tumour, mortality of patients, ER alpha, PR, ERB-2, p53 expression and the presence of p53 mutations.

## DISCUSSION

These results confirm the presence of HPV 18 gene sequences in human breast tumours. The identification of such sequences proved to be difficult and required additional amplification of DNA before PCR and use of SYBR Green I to optimise the detection. These technical difficulties may account for the negative results obtained in several other studies (reviewed in three).

We did not identify HPV types 16 and 33 in these breast tumours. A plausible explanation is that HPV 18 may be present in breast tumours in predominantly Caucasian populations and HPV 33 in breast tumours that occur in predominantly Chinese and Japanese populations (Hennig *et al*, 1999; Yu *et al*, 1999; De Villiers *et al*, 2005).

Five variants of HPV 18 were identified. Variations in the gene sequences indicate that these were not contaminants. Sequence variations were observed in the Sp1-binding site, E2 protein-binding site number 2, between E2 protein-binding site number 1 and 2, a region 12 bp upstream of the first starting codon, and within the translated E6 sequence. As far as we know, these variations are novel and no one has yet studied the effect of these variations on the expression of HPV genes. However, it is anticipated that these variations would not have an impact on the HPV gene expression. This is because those variations do not alter the SP1 and E2 protein-binding motifs, and the base pair change does not alter the E6 amino-acid sequence.

The role of HPV in breast cancer development is not elucidated. As in other studies, the presence of HPV sequences in breast tumour samples is not associated with tumour grade, patient mortality, expression of ER, PR, ERB-2, p53 expression and mutation. Hence, it is not clear if HPV promotes cancer development or the presence of HPV sequences represents a parasitic infection independent of cancer. However, given that HPVs are proven human oncoviruses in cervical cancer, it is unlikely that they act as parasitic infections in human breast tumours.

Human papilloma viruses are mainly transmitted by cell surface contact. Therefore, how are HPVs transmitted to the breast? A suggestive clue comes from the observation by de Villiers *et al* (2005) that HPVs are present in cancers occurring in human nipple milk ducts and that these cancers have the typical histological features of HPV-induced human cancers. In addition, in two independent studies, HPV 16 has been found to be present in breast tumours that occur in European women with HPV 16-associated cervical cancer (Hennig *et al*, 1999; Widschwendter *et al*, 2004). Virions (virus particles) are shed from desquamating keratinocytes (the target cells for HPV infections) and high-risk HPVs can be transmitted by close human nonsexual contact (Bryan and Brown, 2001; Rintala *et al*, 2005). Accordingly, we speculate that HPVs may be transmitted by hand from the female perineum to the breast which could occur, for example, during showering or bathing.

In conclusion, our studies demonstrate the presence of HPV 18 variants in Australian breast cancer specimens. Further studies are required to elucidate the role and pathogenesis of HPV in breast cancer.

## Figures and Tables

**Table 1:** Presence of HPV gene sequences in DNA extracted from 50 human breast tumours

**Correlations of the presence of HPV^a^ with**	**Spearman's correlation coefficient^b^**	**Two-tailed significance^c^**
Tumour grade	0.127	0.378 NS
Patient's mortality^d^	−0.107	0.461 NS
ER-positive tumour^e^	0.132	0.364 NS
PR-positive tumour^f^	0.104	0.479 NS
ERB^g^ receptor	0.089	0.591 NS
p53^h^ expression	0.090	0.533 NS
p53^i^ mutation	−0.103	0.478 NS

a Samples which have HPV sequences identified at least once by PCR are included in the statistical analysis and considered as HPV positive.

b Spearman's rank order correlation for nonparametric data. The correlation coefficient ranges from −1 to 1 for perfect correlations.

c The significance is two-tailed. NS=not significant at 95% level.

d Mortality=death due to recurrence of breast cancer (eight patients).

e ER=oestrogen receptor positively expressed in at least 5% of cancer cells.

f PR=progesterone receptor expressed in at least 5% of cancer cells.

g ERB=ERB receptor expressed in the cytoplasm of cancer cells.

h p53 expression=p53 protein expressed in at least 5% of cancer cells.

i p53 mut=p53 mutations identified in cancer cells.

Correlations with grade, survival, ER, PR, ERB-2, P53 expression and p53 mutations.
